# Bibliometric and visual analysis of spinal cord injury-associated macrophages from 2002 to 2023

**DOI:** 10.3389/fneur.2023.1285908

**Published:** 2023-11-21

**Authors:** Yan Zhao, Qiuqiu Xia, Hui Zong, Yanyang Wang, Huaize Dong, Lu Zhu, Jiyue Xia, Qiming Mao, Zijing Weng, Wenbo Liao, Zhijun Xin

**Affiliations:** ^1^Department of Orthopedic Surgery, Affiliated Hospital of Zunyi Medical University, Zunyi, Guizhou, China; ^2^Institutes for Systems Genetics, Frontiers Science Center for Disease-Related Molecular Network, West China Hospital, Sichuan University, Chengdu, China; ^3^Department of Cell Engineering Laboratory, Affiliated Hospital of Zunyi Medical University, Zunyi, China

**Keywords:** spinal cord injury, macrophages, bibliometrics, CiteSpace, VOSviewer

## Abstract

**Background:**

Spinal cord injury (SCI) triggers motor, sensory, and autonomic impairments that adversely damage patients' quality of life. Its pathophysiological processes include inflammation, oxidative stress, and apoptosis, although existing treatment options have little success. Macrophages have a vital function in controlling inflammation in SCI, with their M1-type and M2-type macrophages dominating early inflammatory effects and late brain tissue repair and regeneration, respectively. However, there is a dearth of rigorous bibliometric study in this sector to explore its dynamics and trends. This study intends to examine the current status and trends of macrophage usage in SCI using bibliometric methodologies, which may drive novel therapeutic options.

**Methods:**

In this study, the Web of Science Core Collection (WOSCC) was utilized to collect publications and reviews on macrophages in SCI from 2002 to 2023. Bibliometrics and visualization analyses were performed by VOSviewer, CiteSpace, the R package “bibliometrix”, and online analytic platforms. These analyses covered a variety of aspects, including countries and institutions, authors and co-cited authors, journals and co-cited journals, subject categories, co-cited references, and keyword co-occurrences, in order to provide insights into the research trends and hotspots in this field.

**Results:**

1,775 papers were included in the study, comprising 1,528 articles and 247 reviews. Our research analysis demonstrates that the number of relevant studies in this sector is expanding, specifically the number of publications in the United States and China has risen dramatically. However, there are fewer collaborations between institutions in different nations, and international cooperation needs to be reinforced. Among them, Popovich PG became the leader in the field, and significant journals include Experimental Neurology, Journal of Neurotrauma, and Journal of Neuroscience. Research hotspots involve macrophage polarization, microglia, astrocytes, signaling, cytokines, inflammation, and neuroprotection.

**Conclusions:**

This analysis gives, for the first time, a comprehensive overview of bibliometric studies on macrophages in SCI over the past 20 years. This study not only gives an extensive picture of the knowledge structure but also indicates trends in the subject. The systematic summarization gives a complete and intuitive understanding of the link between spinal cord damage and macrophages and provides a great reference for future related studies.

## Introduction

Spinal cord injury (SCI) usually leads to motor, sensory, and autonomic deficits, and most patients suffer from paraplegia or tetraplegia, which in turn brings about complications such as lung infections and decubitus ulcers, which significantly affects the quality of life of the patients (1). In addition, interventions targeting macrophages. Various pathophysiological processes are involved in neuronal injury, such as ischemia, oxidative stress, inflammation, apoptosis, etc. Although some research have improved the symptoms of SCI in terms of molecular control, they have failed to improve the repair and regeneration of brain tissues following SCI (2). Within hours after SCI, the creation of associated inflammation and infiltration of immune cells generate more damage to the spinal cord, and preventing the advancement of associated inflammation is crucial to the prognosis of SCI (3). Therefore, clarifying the pathophysiological features of inflammation and immune cell infiltration after SCI provides theoretical guidance for finding new targets and treatments.

In recent years, with the expanding research on the relevant role of macrophages after SCI, Based on their phenotype and function, macrophages are categorized as classic macrophages (type M1) and replacement macrophages (type M2). Type M1 largely mediates the preimmune-inflammatory response, secreting inflammatory substances such as tumor necrosis factor-α (TNF-α) and interleukin-1β (IL-1β), which induce inflammatory responses (4), whereas M2-type macrophages exert anti-inflammatory and repair-regulatory activities and mediate the process of tissue repair and regeneration, which are strongly associated to the anti-inflammatory effects and neural tissue repair after SCI (5). Thus, activation and migration of M2-type macrophages are essential for the removal of cellular waste and demyelinating material, providing the necessary environment for neural axon regeneration, and are key cells in the treatment of spinal cord injuries and the restoration of individual neuromotor function (6). In addition, macrophages produce growth and neurotrophic substances to enhance angiogenesis and neuroprotection (7).

An in-depth understanding of the mechanisms and interactions of macrophages in SCI can help us explore new therapeutic strategies and interventions. By regulating macrophage activity and phenotypic transformation, we can inhibit the occurrence of inflammation after SCI, reduce the damage to neural tissues caused by the inflammatory response, and accelerate the repair process at the injury site. Therefore, interventions targeting macrophages may become a new therapy to promote nerve regeneration and functional recovery, bringing new hope and opportunities for SCI patients. A large number of research articles and reviews related to macrophages in SCI have been published. Still, these pieces of literature only focus on a specific area of macrophages in SCI, such as the involvement of macrophages in the inflammatory role after SCI, and the function of macrophage subtypes. Some aspects of significance, such as the number of publications, the degree of contribution of authors from national institutions in the field, and future research topics and hotspots, have not been systematically elaborated. Additionally, bibliometric analysis is distinct from other types of research articles and review articles as it provides a comprehensive overview of a field's development and trends based on existing literature. It would benefit researchers new to this field of study to be aware of these aspects. However, no literature so far deals with the structure of knowledge and thematic trends of macrophages in SCI.

Bibliometrics is a systematic approach used to evaluate literature within relevant academic disciplines. Through both qualitative and quantitative analysis, this method enables the objective and visual identification of scientific research trends. By providing an overview of the current research landscape and offering insights into future directions of development, bibliometrics plays a crucial role in the field (8). Furthermore, bibliometrics possesses the capability to discern collaborative efforts among diverse authors, institutions, and nations, so enabling the assessment of their scholarly contributions inside a certain academic domain. Researchers usually use a variety of bibliometrics software for visual analysis of bibliometrics, such as VOSviewer (9), CiteSpace (10), and the R package “bibliometrix” (11). In recent years, with the abundance of bibliometric visualization tools and the deepening of related studies, more and more bibliometric studies related to SCI have been conducted; for instance, bibliometric studies on treating SCI with glucocorticoids (12) and stem cells (13). However, there are no bibliometric studies on the role of macrophages in SCI.

Therefore, the aim of this study is to provide a comprehensive bibliometric analysis of the literature on macrophages in spinal cord injury over the past 20 years (2002–2023), including the number of publications, distribution of countries and institutions, collaborative networks of authors, publications in key journals, and themes and hotspots of research. This paper will also assess the limitations of existing studies and explore the directions and prospects for future research. By conducting a bibliometric analysis of studies on spinal cord injury and macrophages, this study is expected to yield a comprehensive overview of the field and provide an important reference for further research and clinical applications. We believe that by gaining a deeper understanding of the role of macrophages in spinal cord injury, we can provide new ideas and methods for the treatment and rehabilitation of spinal cord injury and contribute to the quality of life and functional recovery of patients.

## Materials and methods

### Data source and search strategy

**Figure 1:**
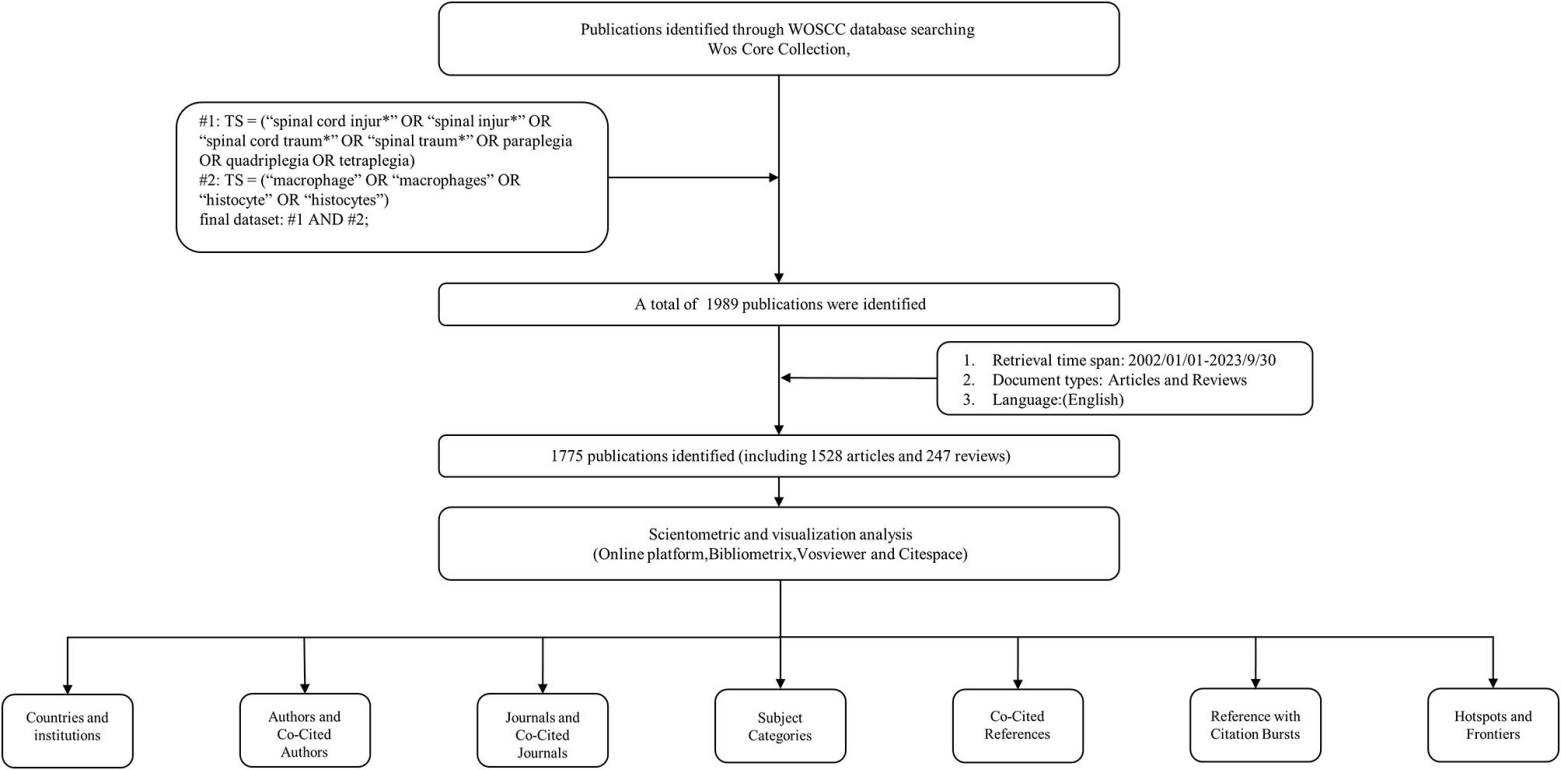
Flowchart of the literature search and screening process.

Web of Science, which contains an extensive collection of literature in the biomedical field, was chosen as the data source for searching the research literature related to SCI and macrophages from January 2002 to September 2023, which was searched on November 30, 2022. The search strategy was:#1: TS = (“spinal cord injur^*^” OR “spinal injur^*^” OR “spinal cord traum^*^ “ OR “spinal traum^*^” OR paraplegia OR quadriplegia OR tetraplegia) #2: TS = (“macrophage” OR “macrophages” OR “histocyte” OR “histocytes”) final dataset: #1 AND #2; A total of 1889 relevant documents were retrieved, and it should be noted that we limited the language of the documents to English, the type of the documents to original articles and reviews, and excluded other contents such as book chapters, letters, biographies, bibliographies, and conference papers, and finally obtained 1,775 documents. The specific screening process is shown in Figure 1.

### Data extraction

According to the search strategy, a total of 1,775 documents were obtained. These documents were exported as “full record and cited references” and saved in plain text format or Win, UTF-8 encoding. We extracted the following data: annual publications, number of citations, country, institution, author, funding agency, journal, subject category, highly cited articles, and keywords. In addition to the above information, we used the “citation report” function in Web of Science to obtain the Hirsch index (H-index) of funding organizations and authors and the average citation index (ACI) of each article. Journal impact factor (JIF) and quartile (Q1, Q2, Q3, and Q4) rankings for subject categories were obtained from the Journal Citation Report 2022 (JCR, Clarivate Analytics, Philadelphia, PA, USA).

### Bibliometric analysis and visualization

For the bibliometric analysis of SCI and macrophages, we used various bibliometric software and tools for data analysis and visualization. First, we performed an visual analysis of the extracted bibliometric data, including annual publications, citations, countries, institutions, authors, funding agencies, journals, subject categories, highly cited articles, and keywords. We used these data to understand the research trends, impact, and distribution of research activities in the field.

Second, the bibliometric data were graphically presented using visualization tools. Using VOSviewer (14), institution analysis, author and co-cited author analysis, journal and co-cited journal analysis, and keyword co-occurrence analysis were generated to demonstrate the citation relationships between institutions, authors, and journals and the hot topics of research, and highly cited institutions, authors, and journals and essential topics of research could be identified by the size and color of the nodes. In addition, this study utilized CiteSpace (15). Institutional analysis, construction of journal bi-graph overlays, analysis of the most relevant topic categories, co-citation analysis of references, and identification of the top 25 references with the strongest citation bursts were carried out to help us discover the connections between the research hotspots and the cutting-edge directions of research.

At the same time, we used the R package “bibliometrix” to construct global distribution cooperation networks and thematic trends in publication volume (16). Another online bibliometric analysis platform (https://bibliometric.com/) for mapping annual publication volume changes and inter-country cooperation networks for the top 20 countries (17); we also used another online platform (https://carrot2.github.io/release/4.5.1/doc/hello-carrot2/) to perform a dendrogram analysis of keywords (18). Bibliometric data analysis and visualization allowed us to gain a comprehensive understanding of research dynamics and trends in the field of SCI and macrophages. These analyses provide valuable information by revealing research hotspots, key issues, and future directions. This information is of great reference value for researchers and policymakers and can guide their research and decision-making.

## Results

### Quantitative analysis of publication

**Figure 2:**
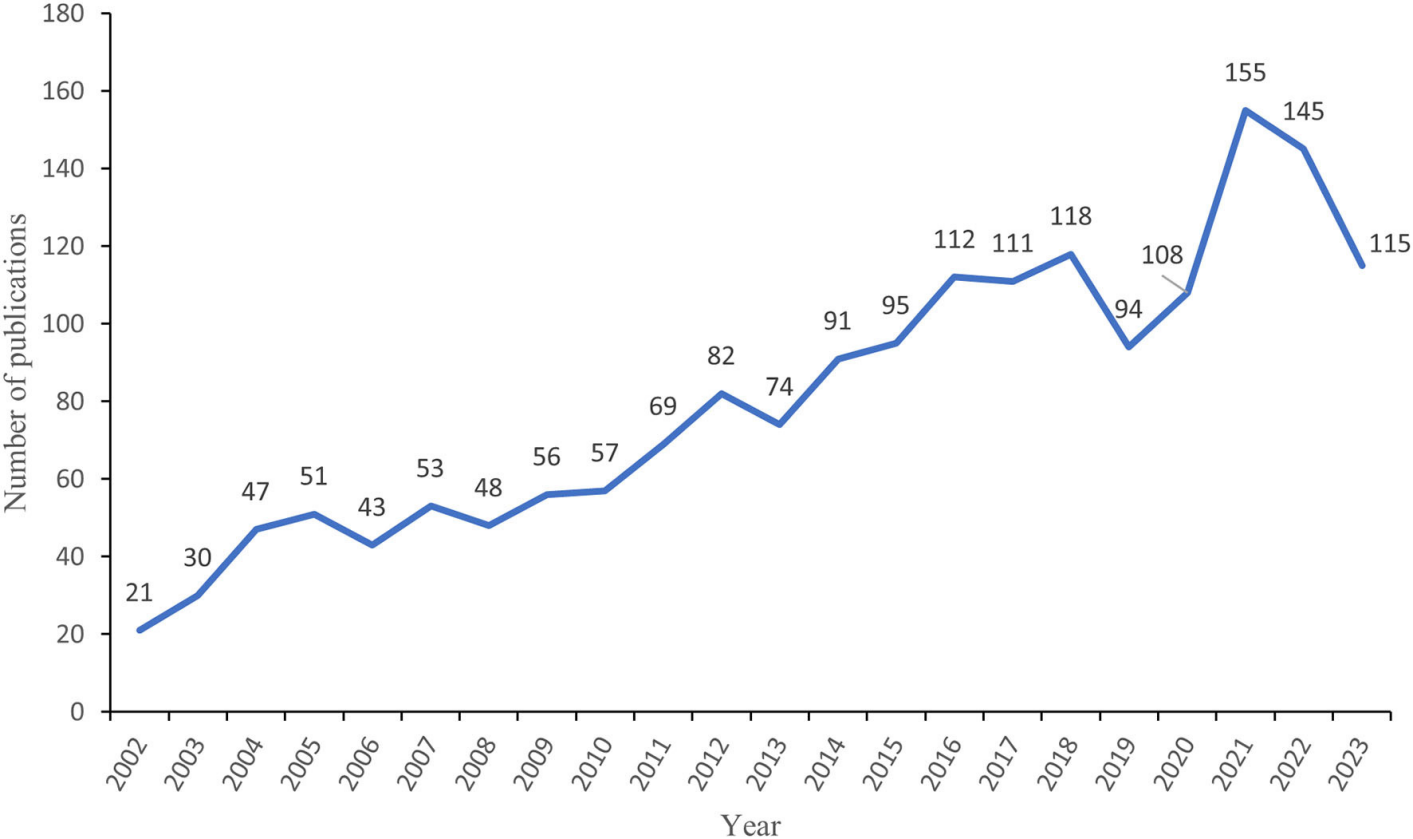
Annual publication volume of spinal cord injury-associated macrophage research.

Based on the search criteria, there were 1,775 eligible publications, including 1,528 research articles and 247 review articles. Figure 2 shows the annual publications and trends in SCI-associated macrophages. Articles in this field have grown progressively globally, especially significantly faster since 2010. However, the number of publications showed an unusual decline in 2018–2019, with possible reasons including a decrease in research funding and the negative impact of epidemics or emergencies on scientific research. The number of publications from 2011 to 2023 was 1,369, or 77.1%. The most significant number of studies was published in 2021, with 155 articles (8.7%). The rapidly increasing number of publications reflects the growing emphasis of researchers on the relationship between SCI and macrophages over the past decade.

### Country and institutional analysis

The publications came mainly from 57 countries and 1609 institutions. Table 1 shows that the top 10 countries in terms of the number of publications are mainly located in Europe, Asia and North America. Amongst this cohort, Europe (*n* = 4), Asia (*n* = 3), and North America (*n* = 2) lay claim to the lion's share. Notably, amidst the entirety of nations, the preponderance of relevant studies found publication in the United States (*n* = 628, constituting 35.38%), with China, Canada, and Germany following in succession. Of the top 10 institutions, the majority were from the China (*n* = 6), followed by United States (*n* = 3) and Canada (*n* = 1). It is worth noting that the United States has the highest H-index and a high ACI in this field, while China has the second highest number of publications but a lower ACI. Meanwhile, Canada performs well in this field with the highest ACI and the second highest H-index.

**Table 1:** Top 10 countries and institutions in SCI-associated macrophage research.

**Rankings**	**Country or agency**	**Volume of publication**	**ACI**	**H-index**	**Total link strength**
1	The United States (North America)	628 (35.38%)	69.19	107	309
2	China (Asia)	525 (29.58%)	24	55	133
3	Canada (North America)	157 (8.85%)	81.37	59	80
4	Germany (Europe)	121 (6.82%)	52.94	43	124
5	Japan (Asia)	113 (6.37%)	46.58	42	43
6	South Korea (Asia)	86 (4.85%)	39.07	33	25
7	Australia (Oceania)	80 (4.51%)	50.94	33	54
8	United Kingdom (Europe)	57 (3.21%)	61.19	29	64
9	Italy (Europe)	49 (2.76%)	70.43	29	33
10	Spain (Europe)	49 (2.76%)	45.43	25	51
1	Ohio State Univ (The United States)	81 (4.56%)	105.68	46	29
2	Univ Miami (The United States)	47 (2.65%)	71.32	27	32
3	Fourth Mil Med Univ (China)	38 (2.14%)	44.42	19	34
4	Nantong Univ (China)	33 (1.86%)	15.36	12	19
5	Sun Yat Sen Univ (China)	32 (1.80%)	24.28	13	29
6	Zhejiang Univ (China)	32 (1.80%)	30.03	17	13
7	Mcgill Univ (Canada)	32 (1.80%)	106.18	27	12
8	Univ Kentucky (The United States)	28 (1.58%)	67.71	21	7
9	Univ Western Ontario (Canada)	27 (1.52%)	70.26	20	5
10	Xi An Jiao Tong Univ (China)	26 (1.46%)	35.27	12	25

**Figure 3:**
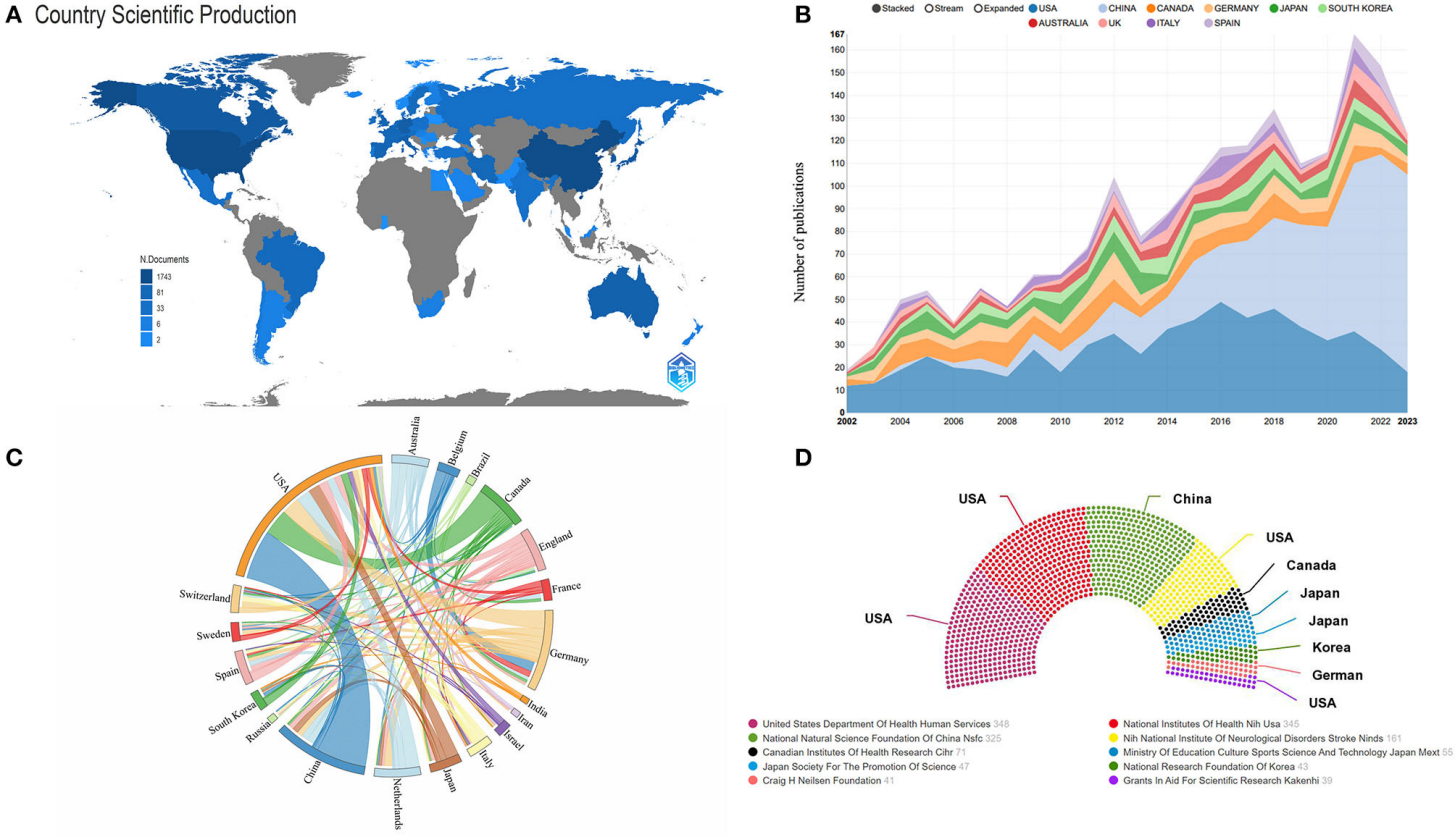
**(A)** Spatial distribution of publications by country. **(B)** Annual number of publications in the top 10 countries from 2001 to 2023. **(C)** Analysis of country cooperation in the top 20 countries. **(D)** Sources of the top 10 funding agencies in the field.

**Figure 4:**
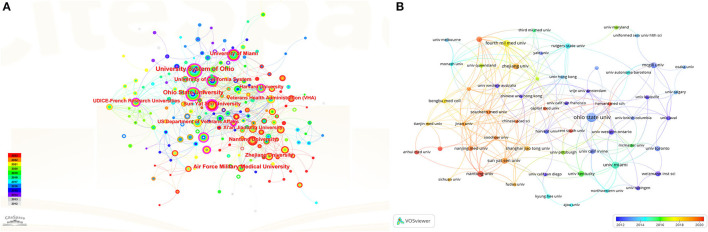
**(A)** CiteSpace-generated institutional collaboration visualization mapping. **(B)** Vosviewer-generated institutional citation volume visualization mapping.

Figure 3A shows the contribution of each country to the total number of publications, with shades of color reflecting the concentration of research, mainly in North America, Asia, and Oceania. Figure 3B illustrates the annual publication volume for the top 10 countries, with the United States dominating from 2002 through 2018 and peaking in 2016. China's publishing volume has also grown significantly since 2014 and has risen even more rapidly from 2018 onwards, gradually overtaking the United States. Figure 3C illustrates international cooperation among the top 20 countries. Figure 3D shows the top 10 funding organizations, with the majority of them coming from the United States (*n* = 4). Figure 4A generates a visual mapping of institutional collaborations through CiteSpace, which shows that Ohio State University publishes the highest number of studies and is at the center of the mapping, with more partnerships with the University Of Miami. Using Vosviewer to filter and analyze the 58 institutions, each of which published at least 12 relevant studies, the three institutions with the largest TLS (Total link strength)were found to be Fourth Mil Med Univ, Ohio State University, and University Of Miami (Figure 4B).

### Authors and co-cited authors

**Figure 5:**
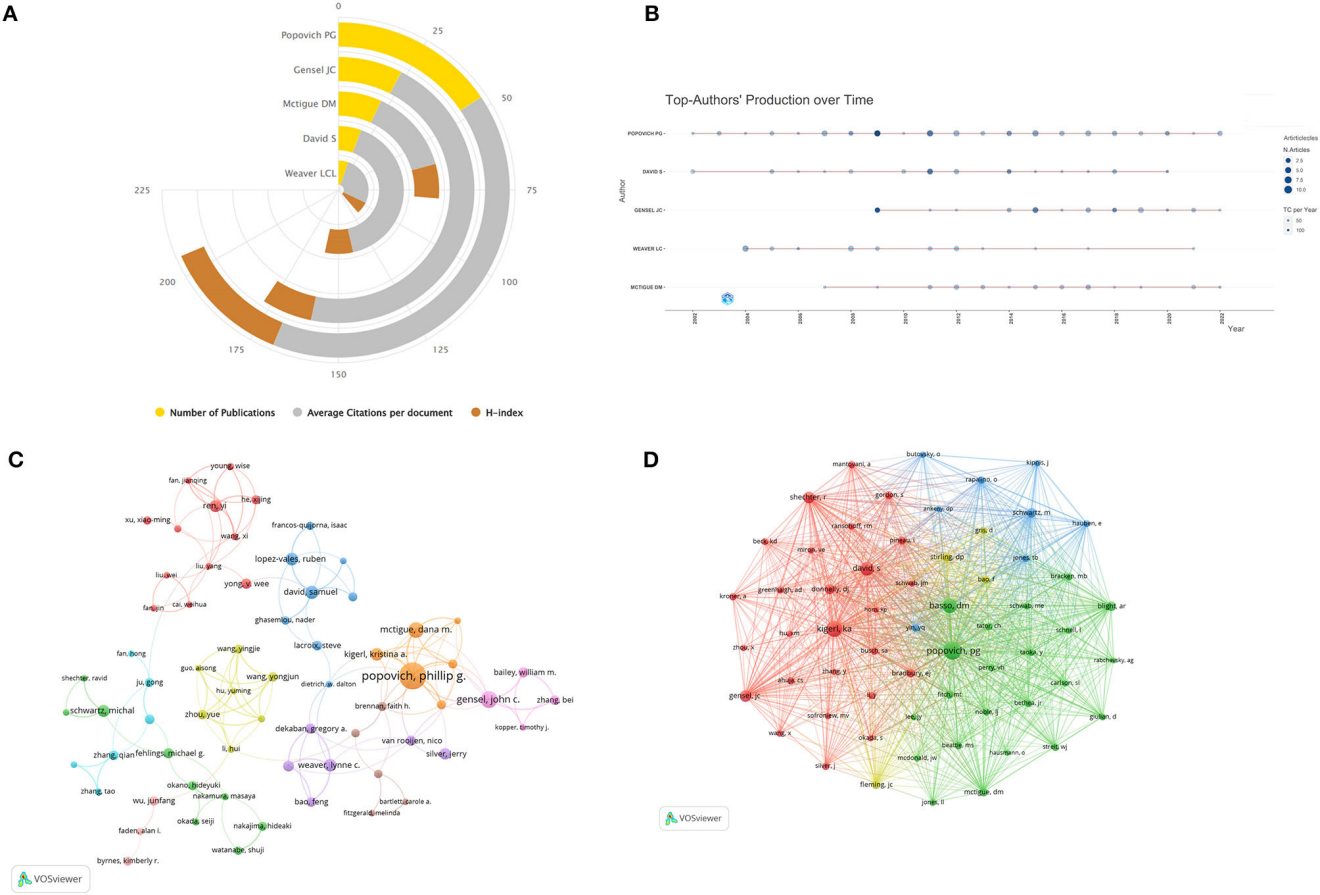
**(A)** Publications, average citations per article, and Hirsch index for the top 5 authors in the field. **(B)** Annual publication and citation counts for the top 5 authors for the period 2001 to 2023; the size of the circle indicates the number of articles, and the depth of the circle indicates the total number of citations per year. **(C)** Author co-authorship analytic network generated by VOSviewer, where authors who work closely together share a common color. **(D)** Co-citation network generated by VOSviewer, where nodes indicate authors, the size of the nodes is proportional to the number of citations, the line in the middle indicates citation relationships and smaller distances between the nodes indicate higher correlations, which are categorized as the same color.

Eight thousand eight hundred and eleven authors were involved in macrophage research related to SCI. Figure 5A shows the top five most productive authors ranked, with Popovich PG publishing the highest number of articles, followed by Gensel JC and McTigue DM. Figure 5B shows the annual publications and citations of the top five authors from 2002 to 2023. We included 66 authors for the study using the number of published articles greater than or equal to 6 as the filtering criterion. We used Vosviewer to create an author collaborative co-authorship network (Figure 5C), which included seven main cluster categories. The orange cluster category, with Popovich PG as the core staff, focuses on macrophage-associated neuroinflammation, neuroprotection, axonal regeneration, and myelin repair after spinal cord injury, as well as the cytokines, receptors, and signaling pathways that influence these processes. The pink cluster category, which collaborates extensively with the orange cluster category, has a research system centered on Gensel JC, which focuses on articles or reviews related to macrophage-associated monocytes, microglia, and neuroprotection after spinal cord injury. And the purple cluster category, with Weaver LC as the core staff, focuses on macrophage-associated oxidative stress after spinal cord injury. The blue cluster category formed a core group of researchers with David S, focusing on the inflammatory response of macrophages from the peripheral circulation and resident microglia after spinal cord trauma, and analyzing their effects on tissue protection and repair, as well as the role of macrophage polarization in nerve regeneration. The red cluster category, mainly centered on Ren Y, explores how myelin debris induces and regulates phenotypic and functional changes in macrophages and microglial cells after spinal cord injury, as well as the effects of these changes on nerve regeneration and inflammatory responses. The yellow cluster category, centered on Wang YJ, focuses on the interactions between neurons and glial cells after spinal cord injury, as well as the effects of some regulatory factors and drugs on nerve regeneration and inflammatory responses. The group centered on Schwartz M in the green cluster category investigated the role and regulation of protective autoimmunity, monocyte-derived macrophages, microglia, and T cells in spinal cord injury, multiple sclerosis, and other CNS diseases, which provided new strategies and methods for nervous system repair. The analysis of co-cited authors refers to the fact that if the articles of two authors are often cited by subsequent studies at the same time, then the research interests or academic ideas of these two authors may be related, and then we consider that there is a co-citation relationship between the two authors (19). By setting the minimum number of citations to 100, we also made the co-citation network (Figure 5D), which shows that Popovich PG, Kigerl KA, Basso DM, and David S are the authors with the highest citation intensity. The network contains three main cluster classes of different colors, the green cluster class with Popovich PG and Basso DM as the main core, the red cluster class contains three authors with high citation intensity such as Kigerl KA, David S, and Gensel JC, and the blue cluster class is mainly represented by Schwartz M as a cluster class with high citation intensity.

### Analysis of journals and co-cited journals

**Figure 6:**
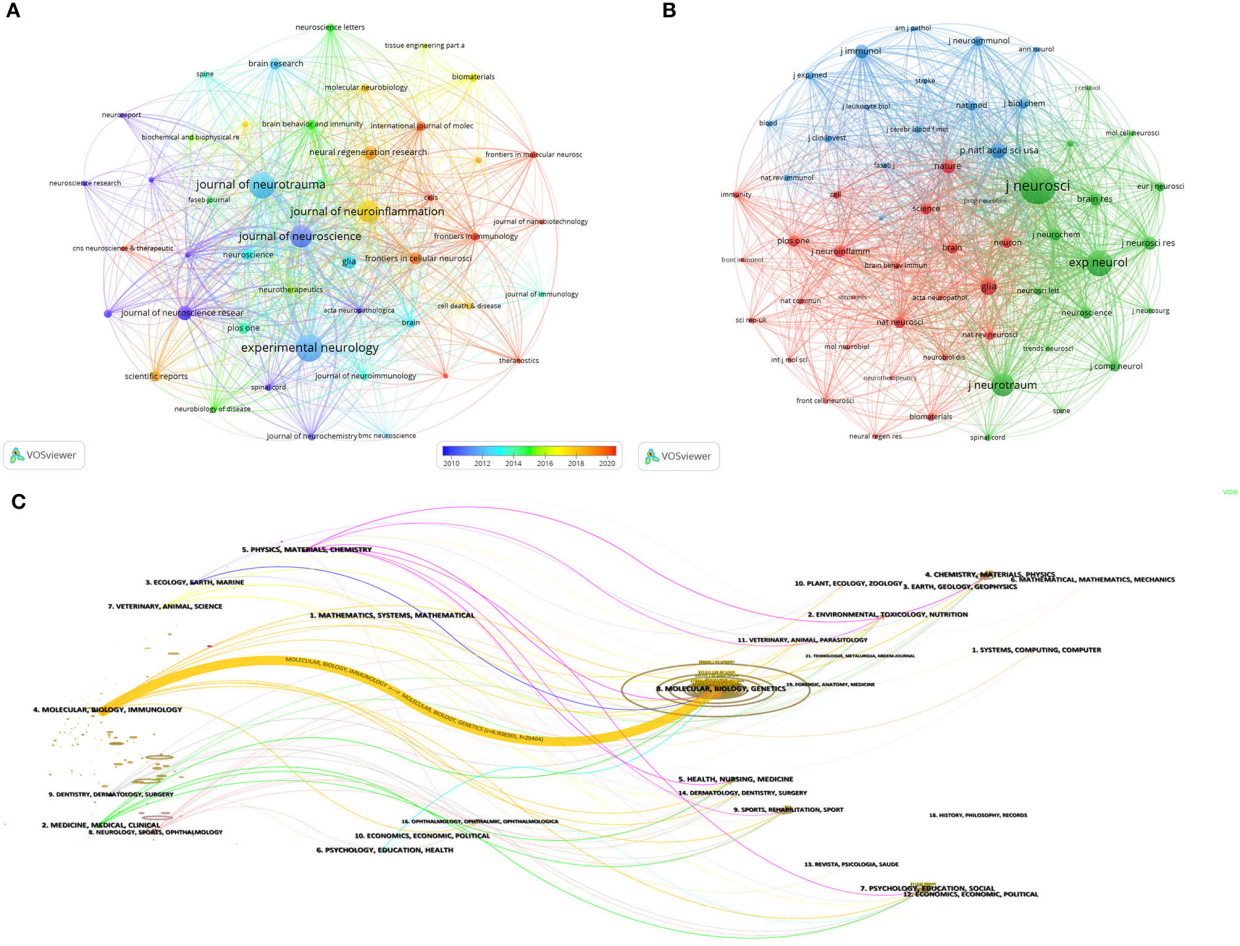
**(A)** Visual network of journals in the field of spinal cord injury-associated macrophage research, node size is proportional to the number of publications, and colors correspond to different years. **(B)** Visual network of journals co-cited in the field of spinal cord injury-associated macrophage research, one node represents one journal, node size is proportional to the number of citations, and the line segments between them indicate citation relationships, and the smaller the distance between the nodes the higher the correlation, and categorized by the same color. **(C)** Dual graph overlay visual mapping of journals in spinal cord injury-associated macrophage research.

Four hundred and eighty six journals published studies related to macrophages in SCI. The top 15 journals with the highest production and most total citations are listed in Table 2, with Experimental Neurology publishing the most articles or reviews (*n* = 87, 4.9%), followed by Journal of Neurotrauma and Journal of Neuroscience. According to the Journal Citation Report 2022, the impact factors of the top 15 journals ranged from 2.9 (Brain Research) to 15.1 (Brain Behavior and Immunity). Subsequently, we selected 38 journals based on the filtering condition of a minimum number of publications of 8 and plotted a visual network of journals (Figure 6A). The figure shows strong citation relationships between journals, especially between Experimental Neurology, Journal of Neurotrauma, Journal of Neuroscience, and Journal of Neuroinflammation, as well as between the four journals centered around these four journals. The citation relationship between the surrounding journals is also strong.

**Table 2:** Top 15 journals and co-cited journals in the field of SCI-associated macrophage research.

**Rankings**	**Journal**	**Counts**	**IF**	**Q (JCR division)**	**Total cited journals**	**Co-citation**	**IF**	**Q (JCR division)**
1	*Experimental Neurology*	87 (4.9%)	5.3	Q1	*Journal of Neuroscience*	10,018	5.3	Q1
2	*Journal of Neurotrauma*	81 (4.6%)	4.2	Q2	*Experimental Neurology*	5,608	5.3	Q1
3	*Journal of Neuroscience*	67 (3.8%)	5.3	Q1	*Journal of Neuroinflammation*	4,315	9.3	Q1
4	*Journal of Neuroinflammation*	65 (3.7%)	9.3	Q1	*Journal of Neurotrauma*	3,158	4.2	Q2
5	*Journal of Neuroscience Research*	36 (2.0%)	4.2	Q2	*Brain*	2,649	14.5	Q1
6	*Neural Regeneration Research*	34 (1.9%)	6.1	Q1	*Glia*	2,044	6.2	Q1
7	*Glia*	33 (1.9%)	6.2	Q1	*Journal of Neuroscience Research*	1,989	4.2	Q2
8	*Frontiers In Cellular Neuroscience*	27 (1.5%)	5.3	Q1	*Biomaterials*	1,888	14.0	Q1
9	*Plos One*	26 (1.5%)	3.7	Q2	*Brain Research*	1,416	2.9	Q3
10	*Brain Research*	25 (1.4%)	2.9	Q3	*Journal of Comparative Neurology*	1,400	2.5	Q3
11	*Neuroscience*	24 (1.4%)	3.3	Q3	*Neurotherapeutics*	1,386	5.7	Q1
12	*Scientific Reports*	24 (1.4%)	4.6	Q2	*Plos One*	1,317	3.7	Q2
13	*Brain Behavior and Immunity*	19 (1.1%)	15.1	Q1	*Brain Behavior and Immunity*	1,045	15.1	Q1
14	*Biomaterials*	19 (1.1%)	14.0	Q1	*Journal of Neurochemistry*	992	4.7	Q2
15	*International journal of molecular sciences*	19 (1.1%)	6.2	Q1	*Frontiers In Cellular Neuroscience*	904	5.3	Q1

Co-cited journals are the phenomenon of two journals being cited in the same literature, reflecting the relevance of various journals and disciplines (20). The analysis of co-cited journals can be used to locate and categorize journals, determine their core or marginal position in the field, and evaluate the importance of academic journals (21). Among the top 15 co-cited journals (Table 2), five journals had more than 2,500 co-citations, with *Journal of Neuroscience* (*n* = 10,018) being the most co-cited journal, followed by *Experimental Neurology, Journal of Neuroinflammation, Journal of Neurotrauma*, and *Brain*. The journal with the highest impact factor was *Brain Behavior and Immunity* (IF=15.1), followed by *Brain* (IF=14.5) and *Biomaterials* (IF=14.0). We set a minimum co-citation of 400 and included 46 journals for mapping the network of co-cited journals (Figure 6B). The top three journals with the strongest TLS in this network were *Journal of Neuroscience, Experimental Neurology*, and *Journal of Neurotrauma*.

Dual graph overlay analysis of journals involves overlaying the co-cited network graphs of journals from two different periods in order to compare the association of journals between the two periods. Figure 6C shows the citing journal clusters on the left and the cited journal clusters on the right, yellow bars indicate the most closely related research areas, and the width of the bar indicates the strength of the citation relationship. The yellow bars indicate that articles published in Molecular/Biology/Immunology journals usually cite articles published in Molecular/Biology/Genetics journals.

### Analysis of the most relevant thematic categories

**Figure 7:**
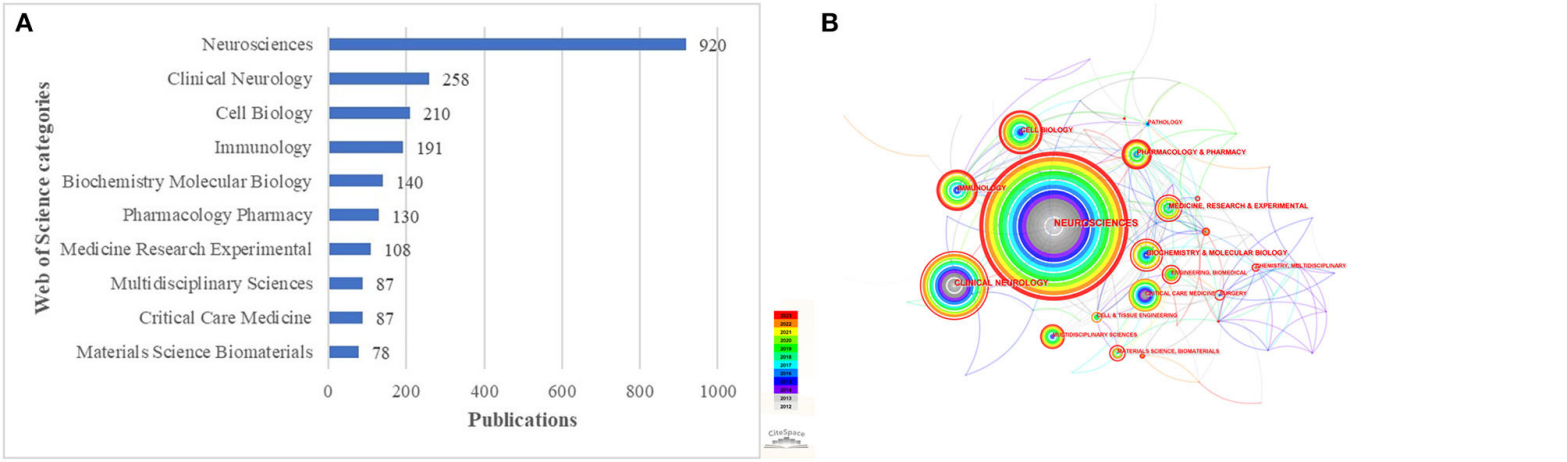
**(A)** Top ten subject categories distributed by number of publications. **(B)** Network of subject categories generated by CiteSpace.

According to the topic category analysis function of the WoSCC database, the top 10 published topic categories can be obtained (Figure 7A). It can be seen that Neurosciences (*n* = 920) related topics have the most research, far more than other research topics. A network of topic categories was subsequently constructed using CiteSpace (Figure 7B), and it can be seen that there is a cross-linkage between the more researched topics, including Immunology, Cell Biology, Clinical Neurology, and Neurosciences.

### Co-cited references and reference burst

**Figure 8:**
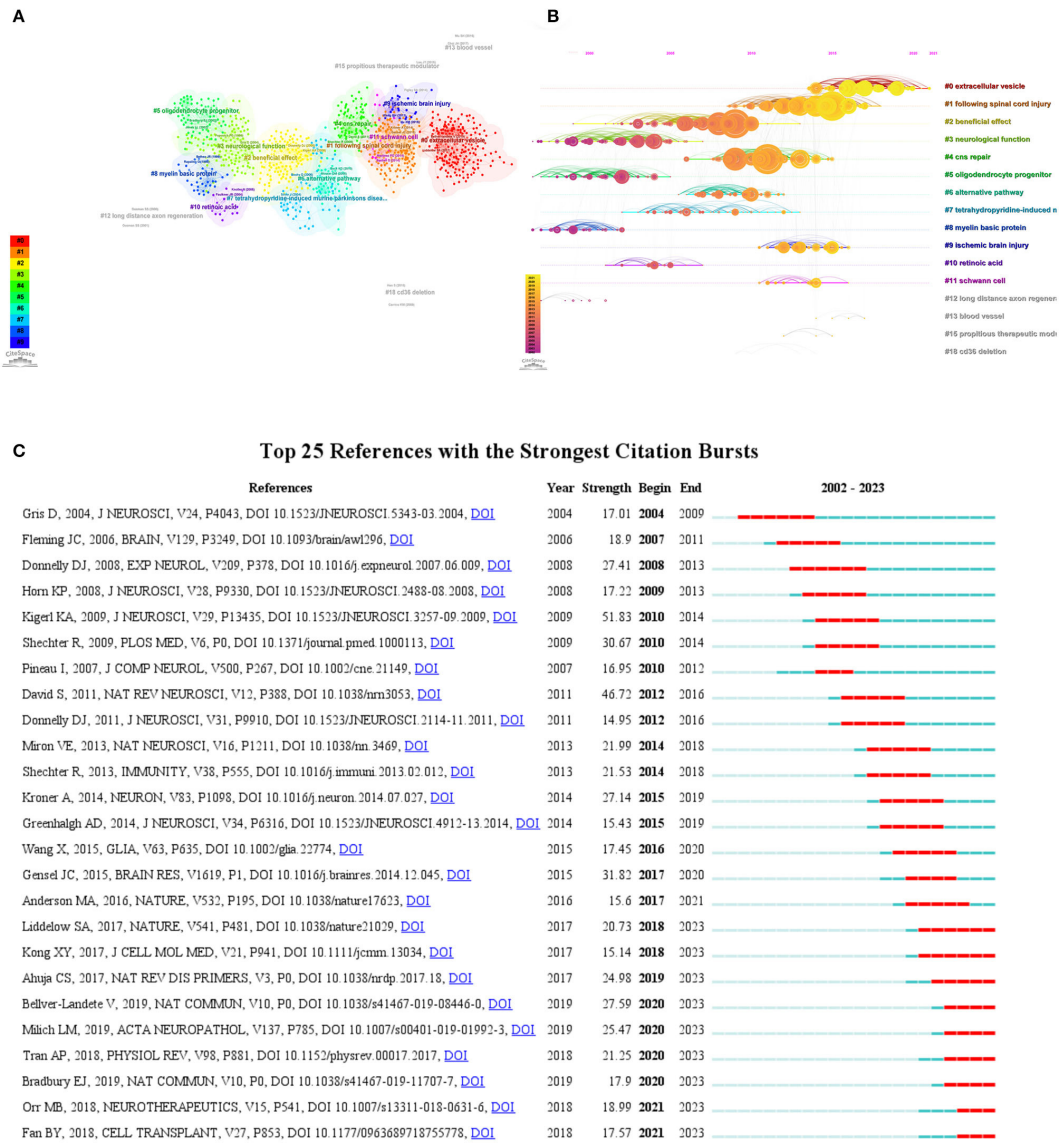
**(A)** CiteSpace-generated clusters of co-cited references, with dots denoting references and different colors denoting different clusters. **(B)** CiteSpace-generated timeline plot of the main clusters. **(C)** The top 25 references with the strongest citation bursts, with red bars denoting the citation burst durations.

We used CiteSpace to analyze co-cited references, as shown in Figure 8A; each dot representing a reference was categorized into different clusters due to the existence of intersecting common research areas, as can be seen in Figure 8A, “extracellular vesicle” (#0) is the largest cluster, followed by “following SCI” (#1), “beneficial effect” (#2) and “cns repair” (#3). Then, the timeline of the major clusters was plotted using CiteSpace (Figure 8B). Based on its temporal direction, it can be seen that the hotspots of research have shifted from the original #2 beneficial effect, #3 neurological function, #5 oligodendrocyte progenitor, and #8 myelin basic protein to #0 extracellular vesicle, #1 following SCI, #4 cns repair, and #9 ischemic brain injury. Table 3 summarizes the information of the top 10 co-cited references, with total citations exceeding 100 and three with more than 200 citations (22–24).

**Table 3:** Top 10 co-cited references in the field of SCI-associated macrophage research.

**Rankings**	**Co-cited references**	**Co-citation**
1	Kigerl KA, 2009, J NEUROSCI, V29, P13435, DOI 10.1523/jneurosci.3257-09.2009	379
2	David S, 2011, NAT REV NEUROSCI, V12, P388, DOI 10.1038/nrn3053	241
3	Popovich PG, 1999, EXP NEUROL, V158, P351, DOI 10.1006/exnr.1999.7118	221
4	Basso DM, 1995, J NEUROTRAUM, V12, P1, DOI 10.1089/neu.1995.12.1	200
5	Basso DM, 2006, J NEUROTRAUM, V23, P635, DOI 10.1089/neu.2006.23.635	185
6	Popovich PG, 1997, J COMP NEUROL, V377, P443, DOI 10.1002/(sici)1096-9861(19970120)377:3 < 443::aid-cne10>3.0.co;2-s	180
7	Donnelly DJ, 2008, EXP NEUROL, V209, P378, DOI 10.1016/j.expneurol.2007.06.009	170
8	Rapalino O, 1998, NAT MED, V4, P814, DOI 10.1038/nm0798-814	160
9	Shechter R, 2009, PLOS MED, V6, DOI 10.1371/journal.pmed.1000113	140
10	Fleming JC, 2006, BRAIN, V129, P3249, DOI 10.1093/brain/awl296	139

Citation bursting in references is the phenomenon where some literature suddenly receives a large number of citations in a specific period (25). Figure 8C illustrates the top 25 literature with the strongest citation bursts, with blue-striped squares indicating the total years and red-striped squares indicating the years of the strongest citation intervals. The first citation burst year is 2002, while the terminal citation burst year is 2023. The duration of citation bursts ranged from 2 to 6 years, and citation burst strength varied from 12.47 to 47.07. We have summarized the main elements of these 25 strongest citation bursts in the literature and listed them in Table 4.

**Table 4:** Main content of the top 25 references with the strongest citation bursts in the field of SCI-associated macrophage research.

**Rankings**	**Strength**	**Main research content**
1	17.01	Treatment with a monoclonal antibody against cd11d/cd18 integrin reduces the early inflammatory response after sci, protecting neural tissue and improving sensory, autonomic, and motor function.
2	18.9	The cellular inflammatory response following human SCI was studied, including the type, timing, distribution, and role of inflammatory cells and the oxidative and protease markers they express to inform anti-inflammatory neuroprotective strategies.
3	27.41	The role of inflammation in neuroprotection, axonal regeneration, and functional recovery after SCI, and therapeutic strategies for targeting neuroinflammation.
4	17.22	Activated macrophages were studied to induce massive contraction of damaged axons in the central nervous system through direct physical interactions, which impedes nerve regeneration.
5	51.83	The distribution and function of different types of macrophages after SCI in mice were investigated, and M1-type macrophages were found to be neurotoxic and axonal growth inhibitory, whereas M2-type macrophages were neuro.
6	30.67	In mice, macrophages in peripheral blood contribute to anti-inflammation and nerve regeneration after SCI.
7	16.95	Exploring temporal and spatial changes and sources of inflammatory and proinflammatory cytokines after SCI.
8	46.72	This article reviews macrophages and microglia in the nervous system after SCI, their role in the repair process, and potential therapeutic approaches.
9	14.95	The neuroprotective role of CX3CR1 signaling after SCI was investigated, and the lack of CX3CR1 signaling was found to reduce inflammation, increase myelin and axon retention, and promote motor recovery.
10	21.99	Investigated how microglia and macrophages promote remyelination in the central nervous system through polarization switching and secretion of activin-A.
11	21.53	It was demonstrated that the cerebral choroid plexus (CP) mediates the directed migration of anti-inflammatory M2 macrophages via VCAM-1-VLA-4 and CD73 to reach the damaged spinal cord, and that this pathway is critical for the recovery of motor function after spinal cord injury.
12	27.14	The effects of TNF and intracellular iron on macrophage polarization in the injured spinal cord were investigated, and it was found that TNF and iron promote M1-type inflammatory responses and inhibit M2-type repair responses, thus aggravating neurological recovery after SCI.
13	15.43	This article compares the differences and effects of microglia and peripheral macrophages in phagocytosis of damaged neurons and tissue debris after SCI; microglia are beneficial for recovery from SCI, while the death of peripheral macrophages may exacerbate secondary damage.
14	17.45	Investigated how bone marrow-derived macrophages are affected by myelin debris after SCI, resulting in altered phenotype and function leading to chronic inflammation and nerve damage.
15	31.82	This study compares and analyzes the similarities and differences between macrophage activation after SCI and macrophage activation in regular tissue repair and explores the role and influence of macrophage phenotype in SCI repair.
16	15.6	Rather than being the culprit that prevents axon regeneration, astrocyte scarring can help axon regeneration under the right conditions. The article also describes promoting axon regeneration in CNS injury by providing growth factors and activating neurons.
17	20.73	Researchers have identified a neurotoxic response to astrocytes (A1) induced by activated microglia that kill neurons and myelinating cells, leading to central nervous system (CNS) damage and degenerative disease, and have revealed possible pathways to stop or reverse A1 formation.
18	15.14	This article reviews the inflammatory response that occurs after spinal cord injury and its relationship to macrophage polarization switching, as well as some of the therapeutic strategies that have been used to achieve neuroprotective effects by modulating macrophage polarization.
19	24.98	A panoramic survey of contemporary breakthroughs and obstacles in epidemiology, pathophysiology, clinical presentations, diagnostics, therapeutic interventions, and preemptive measures for SCI.
20	27.59	Microglia were demonstrated to have an important protective role in astrocyte scar formation, neuronal and myelin cell survival, and motor function recovery by secreting IGF-1.
21	25.47	The spatiotemporal dynamics of macrophage infiltration induced by spinal cord injury are reviewed, the multiple mechanisms of macrophage action during injury repair are analyzed, and the regulatory factors and neuroprotective effects of macrophage polarization status are explored, as well as potential therapeutic targets against macrophages.
22	21.25	In this paper, we systematically analyze the regenerative barriers and success factors after spinal cord injury at the cellular and molecular levels, and introduce some potential therapeutic approaches to promote axonal regeneration and neurological recovery.
23	17.9	This paper reviews the range of responses that occur after spinal cord trauma involving different types of cells and molecules, culminating in a scar that inhibits nerve regeneration and plasticity. The paper analyzes the structure, function, mechanisms, and intervention strategies of the scar and how to achieve a balance between maintaining tissue stability and promoting functional recovery.
24	18.99	The dual role of glial cell activation and inflammatory responses induced by spinal cord injury in nerve damage and repair is reviewed, as well as the experimental and clinical applications and challenges of multiple interventions targeting these responses.
25	17.57	The paper proposes a theory of microenvironmental imbalance after spinal cord injury and analyzes the causes and effects of the imbalance at three levels: tissue, cellular, and molecular, and discusses some possible repair methods.

### Keyword co-occurrence correlation analysis

**Figure 9:**
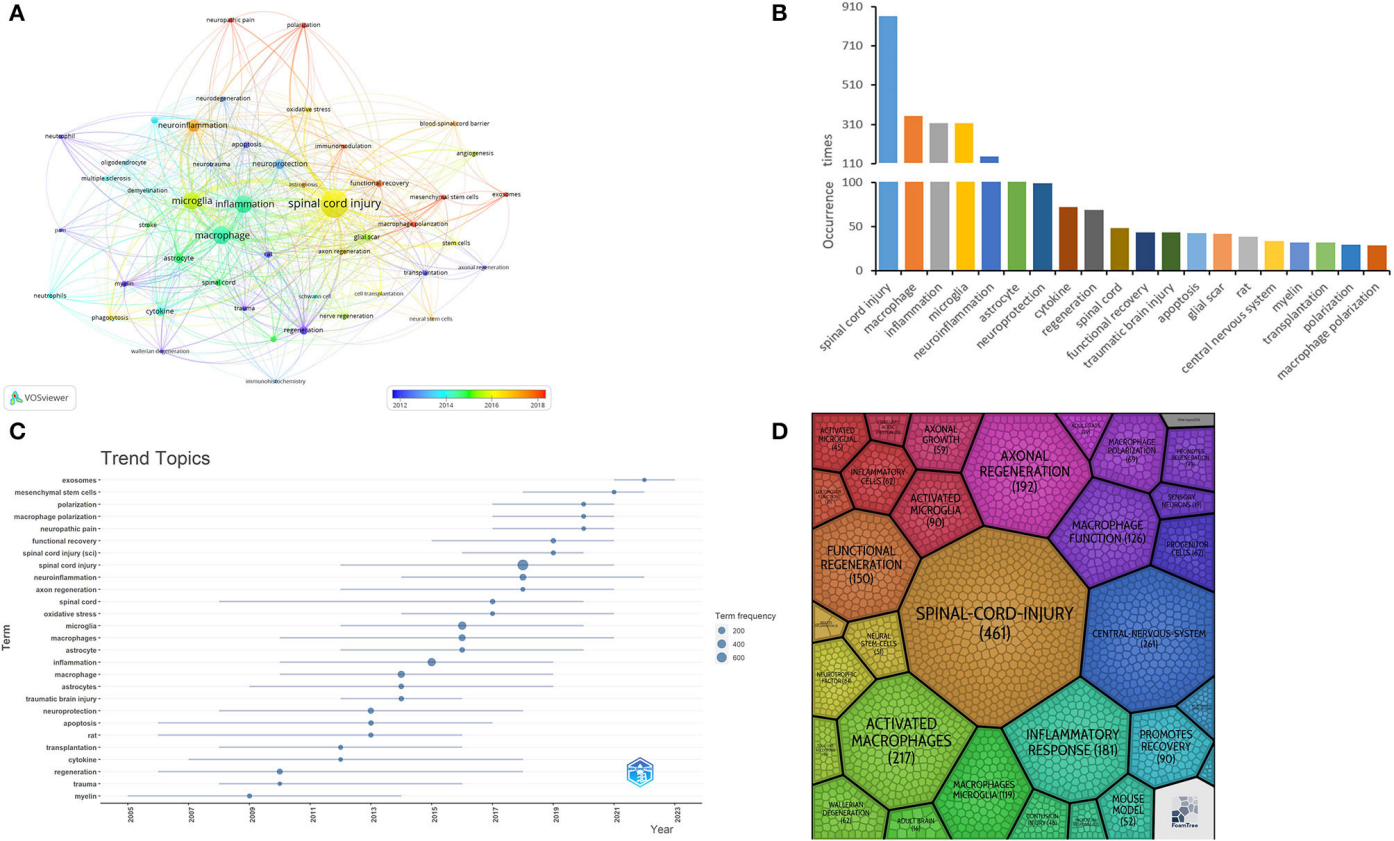
**(A)** Keyword co-occurrence network graph generated by VOSviewer. **(B)** Histogram of a frequency distribution of the top 20 keywords. **(C)** Year-related keywords theme evolution map. **(D)** Keyword dendrogram generated by Carrot2.

We used VOSviewer software to screen the keywords with a frequency greater than or equal to 15. By merging the related keywords of the same significance, we finally obtained 39 keywords and drew the keyword co-occurrence network map (Figure 9A). The hues of the map's dots portray the temporal dimension of research hotspots for the associated keywords, while the dot's dimensions mirror the frequency of the keywords. Interconnecting lines binding these dots signify the intricate relationships amidst the keywords, their thickness indicative of the strength of their interconnection. Evidently, a robust nexus is discernible among SCI, macrophage, inflammation, and microglia. The interconnectedness of SCI, macrophage, inflammation, and microglia is vividly apparent. As depicted in Figure 9B, the graphical representation delineates the frequency distribution of the foremost 20 keywords. Excluding SCI and macrophage, the remaining keywords of notable frequency encompass inflammation (*n* = 293), microglia (*n* = 268), Neuroinflammation (*n* = 112), Neuroprotection (*n* = 94), Astrocyte (*n* = 93), and cytokine (*n* = 64). Additionally, we used the R package to map the keyword theme evolution (Figure 9C), which shows the evolution of topic terms over time, as well as the emergence of new keywords in the domain. In the figure, the size of the dots represents the frequency of the topic words, while the horizontal line indicates the time span of the topic words. As can be seen in Figure 9C, the research theme trends in this field during 2019–2023 were mainly exosomes, mesenchymal stem cells, and macrophage polarization. Whereas, during 2013–2018, the research theme trends in this field were mainly inflammation, oxidative stress, microglia and astrocytes. Prior to 2012, the main focus was on themes such as apoptosis and cytokines. Figure 9D shows the keyword dendrogram generated with the online analysis platform carrot2, which can help researchers identify the core themes and essential keywords in the research field, focus the research direction, and optimize the research design. Table 5 summarizes the 10 keywords with the highest frequency and centrality in SCI-related macrophage research.

**Table 5:** Top 10 keywords with frequency and centrality in SCI-associated macrophage research.

**Rankings**	**Frequency keywords**	**Frequency**	**Centrality keywords**	**Centrality**
1	Spinal cord injury	864	Spinal cord injury	0.28
2	Macrophage	353	Functional recovery	0.16
3	Inflammation	318	Macrophage	0.14
4	Microglia	317	Inflammation	0.13
5	Neuroinflammation	148	Microglia	0.13
6	Astrocyte	106	Regeneration	0.1
7	Neuroprotection	99	Central nervous system	0.09
8	Cytokine	72	Inflammatory response	0.08
9	Regeneration	69	Expression	0.06
10	Spinal cord	49	Apoptosis	0.05

## Discussion

### Overview of SCI-related macrophage research

This paper summarizes research on SCI-associated macrophages using bibliometric methods. From 2002 to 2021, the relevant literature has increased annually, showing that the field has received continued attention from researchers. From 2002 to 2010, a period considered the beginning of the domains, the research literature remained relatively stable. Subsequently, from 2011 to 2015, regarded as the field's developmental stage, the research showed a gradual increase. From 2015, the research in the domains further accelerated, and the number of literatures remained at a high level, marking its entry into a period of rapid development (Figure 2).

Globally, macrophage-related research in spinal cord injury is concentrated in Asia, Europe, and North America (Table 1 and Figure 3A). Of note, the United States has the highest number of articles published in this field and also has relatively high H-index and ACI values, indicating that the United States is leading the way in terms of the quality of research in papers worldwide (Table 1). It was observed that among the top 10 institutions in terms of publication volume, seven research institutions were from the United States, as well as five of the top 10 funding organizations (Table 1 and Figure 3D). This suggests that the United States has strong scientific strength and financial support in spinal cord injury-related macrophage research, forming a network of research institutions centered on Ohio State University, McGill University, and the University Of Miami. In addition, countries such as Canada, Germany, and Japan have also demonstrated high ACI and H-index values in this field, indicating that they also attach great importance to and continue to invest in SCI-related macrophage research. Despite China's rapid increase in the number of publications in this field since 2012, ranking second, its ACI is relatively low compared to other countries. In this case, China's scientific research institutions and researchers will be committed to improving the quality of scientific research, introducing advanced research methods and technologies, improving the quality and influence of papers, and strengthening international cooperation with other countries in the world to make global macrophage-related SCI research achieve more significant results. In order to achieve this goal, Chinese research institutions and scholars can take a series of measures, including strengthening international cooperation, actively introducing advanced research methods and technologies, carefully evaluating research results and controlling the quality of papers, and actively participating in academic conferences and exchange activities. These initiatives are expected to promote the further development of research in this field, enhance the academic level and reputation, and contribute more important scientific results to macrophage-related studies on SCI.

Regarding research authors, Popovich PG published the most articles and had the highest H-index, followed by Gensel JC and Mctigue DM, with Popovich PG and Gensel JC having the highest ACI (Figure 5A). As can be seen in Figure 5B, Popovich PG had the most substantial impact, with the highest number of articles and citations in both 2007–2020, indicating its importance in the field of macrophage-related research in SCI. In addition, the author collaboration analysis graph in Figure 5C shows that the links between individual authors are fragmented, and authors between different countries and institutions should strengthen international collaboration to promote the rapid development of the field of macrophages in SCI. Figure 5D demonstrates the co-citation network of authors in the area, forming a network system centered on Popovich PG, Kigerl KA, and David S. The existence of a dense co-citation network indicates that these authors are widely known and recognized in the field. In one of the most co-cited articles, Kigerl et al. (22) found that after SCI, M1-type macrophages persisted in the CNS and were toxic to neurons, whereas M2-type macrophages were less responsive but protective of neurons and were able to promote the regenerative growth of adult sensory nerve axons, overcoming the effects of inhibitory substances at the site of CNS injury. The results of this study reveal the critical role and regulatory mechanism of macrophages in SCI, which is of great significance for a deeper understanding of the pathophysiological process of SCI and the development of therapeutic strategies.

Regarding published journals, the journals with the highest number of articles were *Experimental Neurology, Journal of Neurotrauma*, and *Journal of Neuroscience*. The vast majority of journals in the top 15 by number of publications were Q1 and Q2 journals, suggesting that the quality of articles in this area is high. Figure 6A shows that Experimental *Neurology* and *Journal of Neurotrauma contributed* the most to the field, and the top 15 journals are likely to be the preferred journals for submissions by researchers in the field related to macrophages in SCI, and that following these journals will help to gain access to the most recent research advances in the area (Table 2). The above journals are also densely cited in relation to each other, forming an extensive network system (Figure 6A). Regarding the co-cited journals, the most cited journal was *Journal of Neuroscience* (*n* = 10,018), followed by *Experimental Neurology* (*n* = 5,608), *Journal of Neuroinflammation* (*n* = 4,315), and *Journal of Neurotrauma* (*n* = 3,158, as well as the top four journals in terms of publications. The citation links between the co-cited journals were also extremely strong (Figure 6B).

### Research hot spots and prospects

Thematic categories in bibliometrics are used to systematically categorize and analyze the different topics covered in the research literature in order to understand better the hotspots, trends, and correlations between topics in the research field (26). Reference analysis examines literature citation relationships, while keyword analysis categorizes and clusters keywords appearing in the literature to reveal hotspots and trends in the research field (27).

As can be seen in Figure 7A, the top five topic categories are Neurosciences, Clinical Neurology, Cell Biology, Immunology, and Biochemistry and Molecular Biology. With the exception of Neurosciences and Clinical Neurology, the other topic categories include Cell Biology, Immunology, and Biochemistry Molecular Biology. Figure 7B shows that Cell Biology, Immunology, and Biochemistry Molecular Biology have strong cross-linkages with other disciplines that may become central themes in SCI-associated macrophage research.

A co-cited reference is a research document in which multiple other documents cite the same one document at the same time (28). This means that these other literatures are associated with that co-cited reference. Studying co-cited references can help scholars understand the hotspots and frontiers of their research fields, discover key literatures of significant academic value, and reveal academic networks and knowledge dissemination in the academic community. The current research hotspots have rapidly evolved toward #0 extracellular vesicle, #1 following SCI, #4 CNS repair, and #9 ischemic brain injury (Figures 8A, B). Among the top 10 co-cited references, Kigerl KA conducted an inquiry into the transformations encompassing macrophage subtypes and functionalities in the aftermath of SCI. Notably, the study discerned the pernicious impact of M1-type macrophages upon neurons, in stark contrast to the restorative role assumed by M2-type macrophages in facilitating nerve rejuvenation, this exploration also encompassed potential avenues for orchestrating the differentiation of macrophage subtypes (22). David and Kroner (23) reviewed the origin, distribution, and phenotype of macrophages and microglia in the CNS after SCI and analyzed their different roles in the acute and chronic phases, including inflammatory response, neuronal protection, axonal regeneration, and scarring, and the article looked at some strategies to utilize the beneficial effects of these cells to treat CNS injuries and diseases, such as drugs, genes, cell transplantation, and biomaterials Popovich et al. attenuated the inflammatory response after experimental SCI and facilitated partial hindlimb functional recovery and neuroanatomical repair by eliminating peripheral macrophages through the use of liposome-encapsulated sodium bisphosphonate chloride (clodronate) (24). Popovich et al. (29) also investigated the cellular inflammatory response, including the distribution, morphology and phenotypic changes of microglia, macrophages, T lymphocytes and astrocytes, in different strains of rats after SCI, and found that strain-specific differences existed, which may be related to the modulation of immune responses by corticosteroids. Donnelly and Popovich (30) reviewed the dual role of different types of neuroinflammatory cells (e.g., neutrophils, macrophages, and lymphocytes) in causing neuronal and glial cell death as well as providing trophic and growth factors, and the authors discussed several therapeutic strategies for targeting neuroinflammation, including cell-specific immunomodulation and pharmacological interventions. Shechter et al. (31) found that a type of monocyte-derived macrophage from peripheral blood had anti-inflammatory and reparative effects after SCI in mice, that these cells had a different phenotype and distribution than microglia residing in the central nervous system and that their number and activity could be modulated by, for example, vaccination or bone marrow transplantation, thus improving functional recovery after SCI. Fleming JC, on the other hand, explored the inflammatory response after SCI in humans, including the temporal and distributional characteristics of different types of inflammatory cells, inflammatory enzymes, and inflammatory regions, as well as their potential impact on damage or repair of neural tissues (32). The content of the above total cited references mainly deals with the role of macrophages and related cells in nerve repair and inflammatory response after SCI. These studies suggest that macrophages and their associated cells play a critical role after SCI. The neurotoxic effects of M1-type macrophages may exacerbate neuronal damage after injury, whereas the promotion of neural repair by M2-type macrophages promises to be a potential target for SCI therapy. Better therapeutic outcomes may be achieved by modulating macrophage subtype differentiation.

Reference citation burst refers to the phenomenon of a significant increase or sudden increase in the number of citations of a particular paper or work in other academic articles in bibliometrics and scientific research (33). This phenomenon indicates that the document or work has significant influence and citation value in a particular field or research direction. Among the main contents of the top 25 references with sudden citation (Table 4), it can be seen that their research hotspots mainly focus on (i) Macrophage type and function: researchers focus on different types of macrophages (M1 and M2 types) and their functions after SCI, and find that M1-type macrophages have neurotoxic effects. In contrast, M2-type macrophages are beneficial to nerve repair. (ii) Inflammation and neuroprotection: the role of inflammatory cells after SCI has been explored in the literature, both in terms of causing neuronal and glial cell death and in terms of providing trophic and growth factors, which can have a meaningful impact on the damage or repair of neural tissue. (iii) Signaling and cytokines: The researchers have focused on the regulatory role of different signaling pathways and cytokines after SCI and explored therapeutic strategies to regulate macrophage subtype differentiation and neuroinflammation. To develop therapeutic and repair strategies, we should focus on precise interventions targeting different types of macrophages to maximize neuronal protection and functional recovery. In addition, the complex role of inflammatory responses after SCI needs to be investigated in greater depth to seek more effective anti-inflammatory treatments. By combining signaling and cytokine modulation, it may be expected to achieve multifaceted therapeutic effects and provide patients with a more comprehensive and effective rehabilitation program.

In keyword analysis, keywords or key phrases are extracted by processing and counting a large amount of literature, articles, or data to understand the topics, research hotspots, and essential contents of SCI-associated macrophages. In this study, keywords with the same meaning were merged, and a keyword network in this field was constructed using Vosviewer (Figure 9A). In Figure 9B, SCI was the primary keyword, and other research hotspot keywords were macrophage inflammation, microglia, neuroinflammation, neuroprotection, astrocytes, and cytokines. Figure 9C demonstrates the evolution of keyword topics between 2002 and 2023, indicating a gradual transition to macrophage polarization and astrocytes. Macrophages, axonal regeneration, inflammatory response, and microglia emerged as key dendritic terms in the keyword dendrogram generated through the bibliometric platform (Figure 9D). In summary, the research hotspots and prospects of SCI-associated macrophages are mainly focused on the following aspects:

#### Macrophage polarization

Macrophages are specialized immune system phagocytes that are extensively activated during SCI. Macrophages are diverse in origin and can develop from bone marrow hematopoietic stem cells or invade from peripheral monocytes to the injury site. During the acute phase of SCI, macrophages exhibit M1-type polarization, and TNF and intracellular iron levels influence the polarization state of macrophages after SCI, which in turn affects neuronal survival and growth, as well as the recovery of motor function (34). However, as the damage progresses, M2-type macrophages begin to predominate, secreting anti-inflammatory factors such as IL-10 and TGF-β, which promote neural tissue repair and regeneration (35). Macrophage polarization is subject to complex regulation, including the influence of intracellular and extracellular factors. Cytokines and chemical signals at the injury site can influence macrophage polarization status. For example, the anti-inflammatory cytokine IL-4, which is mainly produced by infiltrating neutrophils after traumatic SCI, can regulate macrophage activation by inhibiting the production of CCL2, which induces macrophage polarization toward the M2-type direction, thereby limiting secondary tissue damage and cavity formation (36). High doses of lipopolysaccharide (LPS) can promote the recovery of motor function in rats and mice by inducing the production of the neurotrophic factor GDNF by microglia/macrophages in injured spinal cords (37). In addition, epigenetic regulation has a vital role in macrophage polarization. Epigenetic modifications such as DNA methylation can affect gene expression in macrophages, and exercise training can promote functional recovery in spinal cord-injured rats by altering DNA methylation and demethylation in the brain's motor cortex (38).

Secondly, exogenous macrophages can also influence repair after SCI. Researchers found that blood-derived macrophages exerted anti-inflammatory and reparative effects during recovery from SCI in mice, which were dependent on their localization at the site of injury and the expression of interleukin-10, and also demonstrated that this effect was specific and could not be provided by activated glial cells (31). In recent years, developments in genomics and transcriptomics have offered new avenues for unraveling the molecular mechanisms of macrophage polarization. Researchers have explored M1- and M2-type macrophage-specific tracers and factors that influence their polarization, thus contributing to a better understanding of their functions and regulatory mechanisms. This provides new clues for precise interventions targeting macrophage polarization. Shakhbazau et al. (39). Using a novel fluorescent phosphoric acid dendrimer, we explored the polarization state and functional changes of bone marrow-derived macrophages under different conditions, as well as their migratory and neuroprotective roles after SCI, providing new perspectives and approaches for the diagnosis and treatment of neurological diseases and injuries. A study demonstrated that dermal papilla cells from rat beard hair follicles have immunomodulatory properties and can induce the conversion of bone marrow-derived macrophages from proinflammatory M1 type to anti-inflammatory M2 type by co-culture, thus providing favorable conditions for inflammation relief and tissue regeneration after SCI (40). In addition, it has been shown that exosomes secreted by hucMSC can effectively improve motor function in SCI mice, and the mechanism of action may be related to the exosome-induced macrophage conversion from M1-type to M2-type, which inhibits inflammatory response and protects neurons (41).

In conclusion, macrophage polarization's role and regulatory mechanisms after SCI are complex and diverse. An in-depth understanding of the molecular mechanisms of macrophage polarization is expected to provide a basis for developing new therapeutic strategies to promote neural repair and rehabilitation after SCI. However, although some progress has been made, further studies are needed to reveal the deep-rooted mechanisms to provide more effective treatments for SCI patients in the future.

#### Microglia

Microglia are essential members of the central nervous system and play a key role after SCI. They originate from the embryonic neural ectoderm, are formed through differentiation and maturation processes, and are widely distributed in neural tissues. After the occurrence of SCI, microglia's response and regulatory mechanisms have attracted much attention. Studies have shown that microglia are involved in neuroinflammatory, repair, and regenerative processes during both acute and chronic phases of SCI (42). During the acute phase of SCI, microglia exhibit a proinflammatory state, releasing proinflammatory factors such as tumor necrosis factor-α (TNF-α) and interleukin-1β (IL-1β). These molecules mediate the inflammatory response, recruiting and activating other immune cells, such as macrophages and T-lymphocytes, leading to inflammatory injury of neural tissue (43). Some researchers have suggested that M1 microglia produce mainly inflammatory factors and oxidative stressors that lead to neuronal damage and degenerative changes, whereas M2 microglia produce primarily anti-inflammatory factors and neurotrophic factors that promote neuronal survival and regeneration (44). Some drugs or cell transplantation can be considered to increase the number or function of M2 microglia, and the response and function of microglia after SCI are subject to complex regulation, and cytokines and molecular signals at the site of injury can influence the activation status of microglia. Some researchers have used different microglia modulators on microglia responses and axonal degenerative degeneration in an isolated mouse model after laser-induced SCI. Stimulation of microglia with specific Toll-like receptor 2 (TLR2) or TLR4 agonists enhances microglial responses and decreases axonal degenerative degeneration. Th1 cells secrete IL- via IFN-γ-dependent 10, thereby increasing the M2-type microglia/macrophages ratio at the SCI site, producing neuroprotection (45). Other therapeutic interventions, such as microglia-associated inflammation, such as minocycline, PPAR agonists, and anti-inflammatory cytokines, have also become utilized to harness the microglial cell mechanistic response to repair CNS injury (46).

In summary, microglia's response and regulatory mechanisms after SCI are crucial. Their proinflammatory and anti-inflammatory states are alternately involved in inflammatory, reparative, and protective processes, which have a profound impact on the recovery from SCI. However, further in-depth studies on the regulatory mechanisms of microglia are still needed to provide more profound theoretical support for the development of innovative SCI therapeutic strategies.

#### Astrocytes

Astrocytes are widely distributed in neural tissues and originate in the embryonic neural ectoderm. After SCI, the response and functional regulation of astrocytes has attracted much academic interest. Studies have shown that astrocytes play complex roles in different stages of SCI. During the acute phase of SCI, astrocytes are involved in the process of neuroinflammation by releasing cytokines, such as interleukin-1β (IL-1β) and tumor necrosis factor-α (TNF-α). These cytokines are capable of triggering the activation of immune cells, exacerbating the inflammatory response, and may adversely affect neurons and other nerve cells. Brennan FH found that astrocytes have a dual role in the aftermath of SCI through the C5a-C5aR signaling pathway; astrocytes have an injurious effect in the acute phase but a protective or reparative effect in the subacute phase (47). Researchers have found that after SCI, reactive astrocytes die by necrotic apoptosis mediated by receptor-interacting protein 3 and mixed-spectrum kinase structural domain-like proteins, a process that is induced by M1-type macrophages, partly through the TLR/MyD88 signaling pathway (48). However, astrocytes gradually shift to an anti-inflammatory and reparative state with recovery from injury. During the chronic phase of SCI, astrocytes may secrete transforming growth factor-β (TGF-β) to assist in suppressing the inflammatory response and promote repair and regeneration of neural tissue (49). Proliferating reactive astrocytes may also form a scar-like perivascular barrier that restricts the entry and spread of leukocytes into the central nervous system (CNS) parenchyma, thereby protecting CNS function (50).

The response and function of astrocytes are influenced and regulated by a variety of factors. Cytokines and molecular signals at the site of injury have a crucial role in regulating the activation state of astrocytes. For example, inflammatory cytokines such as TNF-α and IL-1β can activate astrocytes and trigger inflammatory responses. Whereas, drugs and tissue-engineered scaffolds can attenuate this effect, Khaing et al. (51) pointed out that the main impact of SCI is the formation of glial scar, which hinders axonal regeneration and repair, while the addition of high molecular weight hyaluronic acid can inhibit the proliferative activation of astrocytes and the production of proteoglycans, which improves the environment of the injury site and provides favorable conditions for nerve regeneration. A physical hydrogel suspension (Chitosan-FPHS) composed of chitosan and water as a scaffolding material can also mitigate this effect and promote axonal regeneration and nerve tissue repair (52). In addition, the interaction of astrocytes with other nerve cells also affects their function. For example, after SCI in rats, simultaneous transplantation of olfactory nerve sheath cells and Schwann cells revealed that this combined transplantation was able to inhibit the activation of astrocytes and macrophages, promote the conversion of macrophages to the M(IL-4) phenotype, decrease the expression of proinflammatory and chemokine factors, and increase the expression of anti-inflammatory factors, thereby improving the immune microenvironment at the site of SCI, and facilitating tissue repair and the recovery of motor function (53). In summary, astrocytes have been found to be a significant contributor to SCI. In conclusion, astrocytes' role and regulatory mechanisms after SCI are complex. They play an essential role in inflammatory response and neuroprotection. However, further in-depth studies are needed to reveal the delicate regulatory mechanisms of astrocytes after SCI, with the aim of providing more clues and rationale for developing more effective therapeutic strategies.

#### Signal transduction and cytokines

After SCI, signaling and cytokines play essential roles in regulating inflammation, repair, and recovery of neural tissues. These signaling pathways and cytokines, derived from multiple cell types, including macrophages, microglia, and astrocytes, collectively participate in a complex regulatory network after SCI. The regulatory mechanisms of SOCS1 and SOCS3 in neuroinflammation after SCI are mediated by intervening in cytokine signaling pathways, regulating the mobilization and activation of microglia, macrophages, and astrocytes, protecting the survival and function of neurons and oligodendrocytes, and have an essential impact on neural repair after SCI (54). It has been reported that CX3CR1 deficiency reduces tissue damage and promotes functional recovery after SCI by reducing the inflammatory and oxidative responses of macrophages in the spinal cord (55). In another study, the authors investigated the effect of macrophage polarization on SCI repair by transiently inhibiting IL-6 signaling via MR16-1 antibody and showed that this strategy increased the number of M2-type macrophages and promoted spinal cord nerve fiber growth and improved motor function (56). In addition, an article reported that ATP promotes the transcription and secretion of CXCL2 by activating the P2X7 receptor on microglial cells, causing the activation of the calcineurin-NFAT and PKC-MAPK signaling pathways, revealing an essential role of CXCL2 in the inflammatory response of the central nervous system (57).

In addition, neuron-glia interactions play an essential role in signaling and cytokine regulation. Chen et al. (58) reported that FGF10 is mainly produced by neurons and microglia/macrophages after SCI and exerts neuroprotective effects through activation of the FGFR2/PI3K/Akt signaling pathway, leading to repair of neural synapses, reduction of apoptosis, attenuation of inflammation, and functional improvement. In neurodegenerative and neuroinfectious diseases, adaptive immunity has an essential impact on neuronal function and survival. Garg et al. (59) found that T cells are able to inhibit oxidative stress-induced neuronal apoptosis by activating astrocytes through the secretion of glutamate, which promotes the release of neuroprotective molecules such as lactate, cysteine, and glutathione. Therefore, an in-depth understanding of signaling and cytokine action mechanisms after SCI is expected to provide new clues for developing precise therapeutic strategies.

In conclusion, after SCI, signaling and cytokines interact in complex ways across multiple cell types to regulate the processes of inflammation, repair, and recovery. Various intra- and extracellular factors, such as cytokine concentration, type, and intercellular interactions, influence cytokine signaling regulation. The role of neurons, which release neurotransmitters that regulate the activation state of glial cells, is also vital in the inter-regulation between different cell types. In summary, the role of signaling and cytokines after SCI and their regulatory mechanisms is a complex and critical research area, and in-depth studies are expected to provide new ideas and methods for the treatment and rehabilitation of SCI.

#### Inflammation and neuroprotection

During the acute phase of SCI, the inflammatory response develops rapidly at the injury site, triggering the activation and signaling of a series of inflammatory cells. Activation of immune cells such as macrophages, microglia, and astrocytes exacerbates the inflammatory response by releasing proinflammatory factors such as tumor necrosis factor-α (TNF-α), interleukin-1β (IL-1β), and interleukin-6 (IL-6). Fleming et al. (32) using immunohistochemical methods to assess the cellular inflammatory response after SCI in humans, including infiltration of neutrophils, monocytes/macrophages, and T lymphocytes, as well as activation of microglia, and to analyze the potentially harmful effects of oxidative and proteolytic enzymes expressed by these inflammatory cells, the results suggest that these inflammatory cells may be involved in neuronal death and axon degeneration after SCI by distinct mechanisms of degeneration. This process results in cell death of neurons, axonal breaks, and abnormal activation of glial cells. However, the inflammatory response is not entirely detrimental, and some degree of inflammation is the body's normal physiologic response to injury, helping to remove cellular waste and guide the repair process. Using a novel cell preparation method and flow cytometry, some researchers have identified a multiphasic inflammatory response with two phases: an early phase (1 day to 3 days) dominated by neutrophils, which primarily perform necrotic tissue clearance and infection defense, and a late phase (7 days to 28 days) dominated by macrophages, which are mainly responsible for tissue repair and regeneration (60). In addition, inflammatory cells at the site of injury also release molecular signals that promote cell survival and regeneration, such as neurotrophic factors, and inflammation has both positive and negative effects on axon regeneration after nerve injury, and different immune cells and molecules are involved in this process, which may help to improve the outcome of nerve injury by modulating the inflammatory response (61). Thus, inflammation is also involved in the process of neuroprotection to some extent.

As SCI progresses, the inflammatory response wanes and enters a chronic phase. During this phase, anti-inflammatory signals gradually emerge, promoting neuroprotection and repair. Increased release of anti-inflammatory factors such as interleukin-10 (IL-10) and transforming growth factor-β (TGF-β) helps to attenuate the inflammatory response and promotes repair and regeneration of neural tissue. However, some studies have also found that there is an effect of age on SCI function and tissue recovery, finding that aging reduces the ability of macrophages to secrete IL-10, leading to a decrease in M2b-type macrophages, which exacerbates the impairment of tissue damage and functional recovery (62). In addition, human bone marrow mesenchymal stem cells (hMSCs) inhibit the inflammatory response after SCI by modulating the expression of the neuropeptide PACAP, and hMSCs were found to enhance the production of IL-4 and promote the activation of M2-type macrophages, which improves neurological recovery after SCI (63). Meanwhile, anti-inflammatory factors also help to regulate the polarization state of glial cells and attenuate the neuroinflammatory response, thus promoting neuroprotection (64).

To summarize, inflammation and neuroprotection constitute a complex dynamic balance system after SCI. The inflammatory response exacerbates injury in the early stages of SCI by releasing proinflammatory factors but also provides essential signals for neuroprotection. Over time, anti-inflammatory signals are progressively enhanced, contributing to repair and regeneration. This complex interaction plays a vital role at the cellular and molecular levels, providing new ideas for the development of more effective therapeutic strategies for SCI in the future.

### Future challenges and reflections

Overall, past studies have provided a deeper understanding of the role and mechanisms of macrophages in spinal cord injury. Some of the contributions made by past studies in the field of macrophages and spinal cord injury are listed below:

I. Defining the role of macrophages: Previous studies have revealed an important role for macrophages in spinal cord injury. Macrophages are involved in regulating inflammatory responses, removing cellular debris and neuronal remnants, and promoting nerve regeneration and repair.

II. Uncovering the functions of macrophage subtypes: Researchers have found that there are different subtypes of macrophages, such as M1 and M2. M1-type macrophages play a role in the inflammatory response, while M2-type macrophages contribute to nerve regeneration and repair.

III. Exploring strategies to intervene in macrophages: Past research efforts have developed strategies to intervene in macrophage function. This has included the use of drugs, growth factors, cell transplantation and gene therapy to modulate macrophage activation and function to facilitate the treatment and rehabilitation of spinal cord injuries.

However, the field of research on macrophages and spinal cord injury still faces a number of problems and challenges:

I. Complexity and diversity: Macrophages are a complex and diverse population of cells whose function and phenotype may be influenced by a variety of factors. Understanding the diversity of macrophages and the mechanisms of subtype transformation remains a challenge.

II. Dual role of the inflammatory response: The inflammatory response plays an important role in spinal cord injury, but excessive or sustained inflammatory responses may adversely affect nerve regeneration and repair. How to balance the role of macrophages in the inflammatory response is a key question.

III. Challenges in cell transplantation and therapy: The utilization of macrophages for cell transplantation and therapy is a challenging task. Issues such as selecting appropriate sources, improving cell survival, and facilitating cell adaptation in an injured environment need to be addressed.

IV. Challenges to clinical translation: Despite some progress in laboratory studies, translating macrophage-associated therapeutic strategies into clinical applications still faces a number of challenges. More clinical studies and rigorous clinical trials are necessary to validate the safety and efficacy of therapeutic strategies.

In summary, previous studies have provided a deeper understanding of the role and mechanisms of macrophages in spinal cord injury, but further efforts are needed to address the related issues and challenges. These efforts could contribute to the development of more effective therapeutic strategies to improve the recovery and quality of life of patients with spinal cord injury.

This study summarizes and discusses the research field of Macrophages and Spinal Cord Injury in depth and in detail, with the limitations mainly due to the current nature of the knowledge and rapid update of the research progress. As scientific research continues to progress, new discoveries and breakthroughs may occur in this therapeutic area, which may include new treatments, new drugs, or new therapeutic mechanisms, etc., and thus some of the most recent findings and therapeutic advances may not have been adequately incorporated. This makes this study somewhat limited in terms of timeliness, and readers need to be alert to new research advances when reading it. As the relationship between mesangial macrophages and spinal cord injury this is still in a phase of continuous development and exploration, its translational clinical applications and effects are not yet fully matured. The results of the clinical trials and therapeutic efficacy covered in this review may be affected by factors such as small sample sizes and deficiencies in study design, and thus more large-scale, multicenter clinical studies are needed to validate their efficacy and safety. In order to overcome the difficulties in the field of macrophage and spinal cord injury research, steps can be taken in the following areas:

I. Multidisciplinary collaboration: Spinal cord injury and macrophage research involves multiple disciplinary fields, including biology, medicine, bioengineering and neuroscience. Promoting cooperation and communication between different disciplines and sharing knowledge and technology will help to gain a deeper understanding of the mechanisms of macrophage action in spinal cord injury.

II. Improvement of animal models: Use of suitable animal models to simulate spinal cord injury and better reflect the characteristics of human spinal cord injury. Improving the design and evaluation methods of animal models can increase the reliability and reproducibility of research results.

III. Development of technological tools: The use of new technological tools and instruments, such as single-cell transcriptomics, proteomics and imaging, allows for a more comprehensive study of macrophage function and interactions in spinal cord injury. The application of these technologies can provide more detailed information at the molecular level and deepen our understanding of macrophage behavior.

IV. Enhancement of clinical research: Strengthen cooperation with clinical research and conduct more clinical trials and observational studies to validate the effectiveness and safety of macrophage-related therapeutic strategies. Combining laboratory research results with clinical practice will help promote the use of macrophages in the treatment of spinal cord injury.

V. Data sharing and cooperation: Establish a data-sharing platform to facilitate data sharing and cooperation among researchers. By integrating and analyzing data from different research teams, the statistical power and reliability of research results can be improved and research progress can be accelerated.

VI. Financial support and policy guidance: Increase investment in research on macrophages and spinal cord injury and provide more financial support. At the same time, relevant policies and guidance should be formulated to encourage and support research in related fields and promote the development of research on macrophages and spinal cord injury.

By taking the above measures, we can promote progress in the field of macrophage and spinal cord injury research, deepen the understanding of the role and mechanisms of macrophages in spinal cord injury, and provide a scientific basis for developing more effective treatment strategies.

## Strengths and limitation

We uphold a unique approach and innovative thinking in exploring the bibliometric study of the interrelationship between SCI and macrophages. First, multiple bibliometric tools and techniques were used to analyze the research in the field in depth and comprehensively. Compared with traditional reviews and meta-analyses, our study provides richer and more diverse information for the area. Second, not limited to a single bibliometric tool, we conducted bibliometric analyses on multiple online platforms to explore the research dynamics in the field of SCI and macrophages from multiple dimensions, making the results more comprehensive and objective. More importantly, we are not limited to quantitative and citation analyses but also apply visualization tools to reveal the research hotspots and prospects, which is unique and more comprehensive than traditional reviews.

However, there are limitations to the study. First, the data were primarily derived from the WoSCC database, perhaps ignoring research from other databases and affecting the comprehensiveness of the results. Given that WoSCC is an authoritative database, although it may not fully reflect the field, it can show the overall research to some extent. Secondly, we only considered the English literature and may have missed results in other languages. However, the main thrust of our study was to explore the overall dynamics of the relationship between SCI and macrophages, and the English literature is highly influential and representative of the field, so this limitation had less impact on the findings.

## Conclusion

Taken together, bibliometric studies addressing the relationship between SCI and macrophages reveal key data trends and characteristics in the field. The findings indicate that macrophage-related SCI research has shown a gradual increase over the past few years, with the United States and China, in particular, contributing more significantly to the scientific field. In addition, the United States has the largest number of funding agencies and research institutions. The study also found that there is some fragmentation of collaboration between different research institutions and authors, which raises the need for international cooperation for future in-depth exploration of research. Popovich PG emerged as a critical author leading the field, while the authors represented by *Experimental Neurology, Journal of Neurotrauma*, and *Journal of Neuroscience* are the journals that have become more influential carriers. Co-occurrence analysis of keywords showed that concepts such as macrophage polarization, microglia, astrocytes, signaling and cytokines, inflammation, and neuroprotection were hot topics of research. This phenomenon not only highlights the critical role of macrophages in SCI but also provides a valuable reference for future research directions. In addition, studies have covered new areas of SCI treatment, such as gene therapy and cell transplantation, highlighting the keen interest of the academic community in developing innovative therapeutic strategies. The in-depth bibliometric analysis provides a comprehensive understanding of the current status and future trends of SCI and macrophage-related research. This will provide valuable information to researchers to guide them in more in-depth explorations in this field and provide valuable insights for the development of innovative therapeutic strategies.

In summary, past research has deepened our understanding of macrophages' roles and challenges in spinal cord injuries. Efforts are needed to address the field's complexities, such as macrophage diversity and the dual role of inflammation. Challenges in clinical translation and the dynamic nature of research require rigorous clinical studies. Furthermore, enhancing multidisciplinary collaboration, refining animal models, utilizing advanced technologies, intensifying clinical research, and fostering data sharing are key strategies for advancing macrophage and spinal cord injury research. These measures aim to provide a scientific basis for more effective therapeutic strategies, ultimately improving the quality of life for spinal cord injury patients.

## Data availability statement

The original contributions presented in the study are included in the article/supplementary material, further inquiries can be directed to the corresponding author.

## Author contributions

YZ: Writing—original draft. QX: Writing—original draft. HZ: Writing—original draft. YW: Writing—original draft. HD: Writing—original draft. LZ: Writing—original draft. JX: Writing—original draft. QM: Writing—original draft. ZW: Writing—original draft. WL: Writing—review & editing. ZX: Writing—review & editing.
